# The Surgery of the Pancreas

**Published:** 1887-04

**Authors:** 


THE SURGERY OF THE PANCREAS.

The most important surgical paper of last year, in this country at least, was read by Dr. Nicholas Senn, of Milwaukee, before the American Surgical Association at its last annual meeting, and has now been issued as a reprint from the "Transactions of the Association." Those who are familiar with former papers of Dr. Senn, with the records of his careful experiments, and with his lucid style, need not be told more as to the importance of the paper. But whether our readers be or be not familiar with his writings, they will at once recognize that a carefully written paper of 129 octavo pages on the surgery of the pancreas must contain much valuable material, and positive additions to our knowledge of this very obscure subject. The surgery of the pancreas has no history except as to the treatment of a few cases of cysts of the gland, but the results in these cases have been so encouraging that there is much hope for the future of the surgery of the organ.

The experiments of Dr. Senn as to complete section of the pancreas tend to show that this lesion, unless complicated by other and more serious lesions, is not dangerous to life if the chief source of danger, hæmorrhage, be properly treated. "The co-aptation of the divided ends would be desirable, but is not essential, as the continuity of the duct is not restored after this injury." No disturbance of digestion was noted in either of the two experimental cases, since a sufficient amount of pancreatic juice was secreted from that portion of the organ in communication with the intestine. In case of this injury the most important indication is to arrest bleeding, and to suture the two cut ends of the organ so as to keep them in place, and thus maintain normal blood-supply. In an experiment with regard to the effect of laceration of the pancreas death resulted from the accidental re-opening of the abdominal wound. "Hæmorrhage was arrested spontaneously, and the process of repair, so far as the wound in the pancreas was concerned, appeared to be satisfactory. The divided ends were displaced considerably immediately after the laceration, but were subsequently brought into close contact by the cicatricial contraction." Believing that putrefaction cannot occur without specific germs, Dr. Senn crushed the pancreas of a cat to the extent of two inches in order to test the matter. The animal lived until it was killed, on the eighty-sixth day. It was found that the crushed portion was removed by absorption, which seems to be very rapid in this locality, and which may be explained by assuming that the peritoneum takes an active part in the process. No infection took place, nor could any evidence of putrefaction be found. "Subsequent degeneration, atrophy and sclerosis, take place in that portion of the gland which is no longer connected with the intestine by a permeable duct."

It was found that complete extirpation of the pancreas is invariably fatal, though Schiff has asserted the contrary; and it is important to know that in the two specimens from experiments which showed evidence of gangrene, this was seen on the convex surface of the bowel, but did not in either case involve the entire diameter of the intestine. Surgically speaking, partial extirpation of the pancreas is less serious than complete removal, though physiologically the consequences may be the same if the portion removed embrace the common duct or the two principal ducts from the two portions of the gland. In all of Dr. Senn's experiments the common duct was removed with the excised portion, and thus left the animal, physiologically, without a pancreas. In two cases the animals lived long enough to show the influence of the pancreatic juice upon digestion and assimilation. In each of these animals "the general health and nutrition remained unimpaired for four weeks, when emaciation, with fatty stools, followed, which resulted in death from marasmus in one, after seventy days, and reduced the second to a skeleton in 126 days." As from the beginning no pancreatic juice passed into the intestine it is difficult to account for the good condition of the animals for the first four weeks. Had the marasmus been due to the resection of the mesentery of the duodenum, by diminution ot intestinal absorption, it should have begun earlier. We may assume that the pancreatic tissue left continued to secrete until it was incapacitated by degenerative changes; and with this assumption we must believe that the pancreas could absorb its secretion, and that this, entering the circulation, must have had some influence upon digestion. It is also important to know that in one case almost the whole duodenum was suddenly deprived of its blood-supply, and yet gangrene did not occur, as collateral circulation was set up by the development of two vessels in a band of cicatricial tissue along the concave surface of the bowel.

Seventeen experiments were made to prove the feasibility of ligation of either portion of the pancreas near the common duct as a surgical measure, and the regularity with which the pancreatic tissue is removed by degeneration and absorption of the detached portion of the gland. The ligatures used were of rubber. In every case in which complete physiological detachment was produced by the ligatures, resection, crushing, or any other means, atrophy followed. In no case was anything like a cyst produced. The facts of this atrophy and the non-formation of cysts showd that "in operations upon the pancreas it is not essential or necessary to remove peripheral portions of the gland, for fear that if any of the parenchymatous structure should remain a retention cyst would follow. In partial resection for injury or disease it would be advisable to ligate the peripheral portion, and permit it to remain, as it would lessen the danger by the infliction of less traumatism, and we can confidently expect that it will be removed in a short time by absorption." So also the important pathological question of retention cysts is settled by the experiments; such cysts cannot be due to obstruction of the duct. Again, we are taught the importance of removing those portions of the organ which are not supplied with blood-vessels rather than to trust to absorption, as dead pancreatic tissue is very putrescible.

Experiments were made for the purpose of studying detached portions of the pancreas, and the method of operating in these cases is worthy of the attention of physiologists, though it need not be detailed here. The experiments showed conclusively that when a portion of the pancreas is separated secretion continues until complete degeneration and absorption have caused the disappearance of the parenchyma. "That the atrophy in the part of the organ which has been detached from its connections with the intestine is not due to a traumatic interstitial pancreatitis is proved by the normal appearance and structure of the remaining portion of the gland which has retained its anatomical and physiological relations to the intestine": which again supports the author's view that physiological detachment of any portion of the pancreas is invariably followed by degeneration and complete atrophy, consequently also by complete cessation of functional activity. Eleven experiments were made for the purpose of determining the action of the pancreatic juice on the peritoneum. In only two of the cases was death caused by purulent peritonitis, and the results justify the conclusion that normal pancreatic juice does not cause peritonitis when brought into contact with the peritoneum; and the further conclusion that such juice is absorbed. The duodenum in these cases was denuded of its mesentery, and thus of its vascular supply, for from one to three inches, but no gangrene occurred. One experiment showed that even if no pancreatic juice be produced, digestion may remain good; and the ligation experiments show that the introduction of normal pancreatic juice into the circulation is not harmful, and may be tolerated for two or three weeks without bad consequences.

It has already been seen that crushing and laceration of the pancreas are not of themselves necessarily fatal; and if, during an exploration for abdominal injuries, this organ be found extensively crushed, "it would be good surgery to remove the crushed portion after preliminary ligation of the organ on each side of the comminuted portion. Ligation of the pancreas can be safely done with a single catgut or silk ligature, as the friable texture of the organ will permit of burying the ligature deeply." Crushed pancreatic tissue and pancreatic juice must be removed to prevent traumatic infection. In a case of prolapse of the pancreas, if the prolapse be recent and there are no signs of inflammatory or other changes, it should be thoroughly disinfected and replaced with great gentleness; and if reduction be difficult the wound should be enlarged. Should the organ be in an inflammatory or gangrenous condition "the parts should be thoroughly disinfected and the organ pulled further into the wound until healthy tissue is reached, when a ligature is applied and the diseased portion removed with the knife or scissors. After thorough disinfection the stump is dropped into the abdominal cavity and the external wound closed. Thorough primary removal of infected tissue is the only safety against subsequent extension of the infection to the peritoneal cavity, and the only guarantee for primary union of the abdominal wound." Gangrene, one of the terminations of acute inflammation of the pancreas, may also be included among the diseases of the pancreas which may be treated by surgical measures; inasmuch as spontaneous recovery has followed the elimination of the necrosed organ, it seems not unlikely that timely removal of the necrosed pancreas would add to the chances of recovery. It should not be forgotten that the pancreas may be a part of the intussusception in a case of invagination, and in abdominal section for the relief of this condition the pancreas should not be overlooked. In searching for the cause of this condition, or that of peritonitis, when it is found that the primary disease is located in or around the pancreas radical measures should be adopted when practicable. "Whenever the sac can be stitched to the external incision this should be done, and the sac opened, disinfected, and drained. Search should be made for the necrosed pancreas, and when found detached it should be removed. As in most of these cases the retroperitoneal tissue is extensively infiltrated, a counter-opening should be made in the lumbar region above the kidney, and thorough drainage established. If an anterior abdominal fistula cannot be established, the course to be pursued should be the same as in treating a pancreatic abscess under similar conditions."

In a subsequent issue we will notice that portion of this valuable paper which deals with other pathological conditions of the pancreas.—*Journal of the American Medical Association.*

EXPERIMENTS IN TRAP-SIPHONAGE AT THE NAVY MUSEUM OF HYGIENE.

Repeated experiments on trap-siphonage have shown beyond question that the ventilation of soil-pipes and traps is a safeguard against the entrance of sewer gas into dwellings except in unusual contingencies, provided the general plan of the drainage and sewerage is correct and the workmanship good. The experiments at the Navy Museum in Washington have confirmed the results of those reported to the National Board of Health by Philbrick and Bowditch, and are stated in the following terms:

1. The seals of ventilated traps are safe against siphonage and back pressure.

2. The seals of unventilated traps are never safe from siphon action or back pressure, except in deduction four.

3. The vertical vent should be three inches, with a four-inch soil-pipe.

4. Traps connected on a horizontal pipe and fixtures discharging on the same level into horizontal pipe, apparently have no effect on unventilated traps.

5. All varieties of non-mechanical traps are more easily affected by back pressure than by siphonage.

6. The ball traps were not affected by back pressure, but by siphonage.

7. The Sanitas trap withstood siphon action better than any of the patent traps, but was easily affected by back pressure.

8. The sewer air is more liable to enter unawares by back pressure through the seal of the trap, because the seal remains unbroken.

9. Difference in friction of iron and lead pipes made no apparent difference in the effect on the traps.

We are left in the dark, however, as to the precise nature of the unusual contingencies under which it is admitted that ventilated traps may be siphoned out and the emanations from the sewers enter houses; and Putnam and Waring think that they have shown that the so-called unusual conditions may occur where not in the least suspected, although a patent trap and water-closet intended to meet these "unusual contingencies" might perhaps serve as a bias against entirely unprejudiced judgments in the case.

It is true, however, that the approved methods of plumbing are now so costly as to make them practically unattainable to many householders, and that, outside of a very simple system of plumbing for moderate-sized houses, it is difficult to know with certainty what is safe and what is not. The Museum of Hygiene, under the Navy Department, has an excellent opportunity to show just what the conditions are by which water-closets and soil-pipes inside a house may be made free from danger, and it is to be hoped that this will be done, no matter what the cost of time or money. If plumbing can be simplified and cheapened at the same time, the benefit to society will be incalculable.—*Medical News.*

